# Mechanisms of Effector-Mediated Immunity Revealed by the Accidental Human Pathogen *Legionella pneumophila*

**DOI:** 10.3389/fcimb.2020.593823

**Published:** 2021-02-03

**Authors:** Tshegofatso Ngwaga, Deepika Chauhan, Stephanie R. Shames

**Affiliations:** Division of Biology, Kansas State University, Manhattan, KS, United States

**Keywords:** effector-triggered immunity, effector-mediated immunity, *Legionella pneumophila*, innate immunity, macrophage

## Abstract

Many Gram-negative bacterial pathogens employ translocated virulence factors, termed effector proteins, to facilitate their parasitism of host cells and evade host anti-microbial defenses. However, eukaryotes have evolved to detect effector-mediated virulence strategies through a phenomenon termed effector-triggered immunity (ETI). Although ETI was discovered in plants, a growing body of literature demonstrates that metazoans also utilize effector-mediated immunity to detect and clear bacterial pathogens. This mini review is focused on mechanisms of effector-mediated immune responses by the accidental human pathogen *Legionella pneumophila*. We highlight recent advancements in the field and discuss the future prospects of harnessing effectors for the development of novel therapeutics, a critical need due to the prevalence and rapid spread of antibiotic resistance.

## Effector-Mediated Immunity Enhances Host Defense Against Bacterial Pathogens

The evolutionary arms race between host and pathogen has necessitated the use of several complementary innate immune pathways to detect and eradicate pathogens. Initial pathogen recognition occurs through engagement of pathogen associated molecular patterns (PAMPs) by host pattern recognition receptors (PRRs) (Janeway, 1989). PRRs include toll-like-receptors (TLRs), located on either the plasma membrane or endosomal membranes (Medzhitov and Janeway, 2000; Massis and Zamboni, 2011). PRR recognition of PAMPs activates signaling cascades that culminate in production of pro-inflammatory cytokines that contribute to controlling infection (Janeway and Medzhitov, 2002). Inflammasomes are multimeric intracellular protein complexes that activate inflammatory caspases in response to cellular damage or pathogen infection [reviewed in (Martinon et al., 2002)]. Bacterial pathogens have evolved diverse repertoires of virulence factors to promote their survival within hosts by acquisition of host-derived nutrients and avoidance of host defenses. Bacterial effectors are directly injected into host cells through specialized secretion systems and functions within the host cells to facilitate pathogen survival in close association with host cells (Cambronne and Roy, 2006; Galán, 2009). Both intracellular and extracellular pathogens utilize effector proteins, emphasizing their importance in the virulence strategies of diverse bacterial pathogens.

Multiple effector-mediated virulence processes are similar between seemingly diverse bacterial pathogens. Multicellular eukaryotes are able to detect bacterial effectors and/or their virulence processes *via* effector-triggered immunity (ETI) (Stuart et al., 2013; Rajamuthiah and Mylonakis, 2014; Fischer et al., 2019). ETI was first described in immune defense against pathogens in plants as “gene-for-gene resistance” where resistance (R) genes in plants recognize bacterial effectors (Avr) within the plant cell and trigger an immune response (Flor, 1971; Chisholm et al., 2006). However, animals also detect pathogen infection through ETI and effector-mediated responses [reviewed in (Fischer et al., 2019)]. Plant ETI results from either direct recognition of the effector itself or sensing of intracellular effector activity, whereas only the latter has been observed in metazoans (Stuart et al., 2013). In plants, several models have been described of how resistant strains directly or indirectly detect pathogen effectors. The “receptor-ligand model” describes direct recognition of bacterial effectors whereby a host R protein binds and inactivates a bacterial Avr effector (Stuart et al., 2013). The *Pseudomonas syringae* effector AvrPto blocks host pathogen recognition through subversion of receptor-mediated signaling. In resistant plants, AvrPto is directly inactivated by the R protein, Pto (Xiang et al., 2008). The “guard hypothesis,” “decoy model,” and “bait-and-switch model” are indirect models through which resistant plants detect pathogen effector function (Dangl and Jones, 2001; Stuart et al., 2013). In animal cells, effector function is detected indirectly though cell-autonomous sensing of homeostatic perturbations elicited by the effectors to the benefit of the pathogen (Colaço and Moita, 2016). Examples include cellular detection of effector-mediated translation inhibition, inhibition of Rho GTPases, and pore formation (Fischer et al., 2019). This mini review is focused specifically on mechanisms of effector-mediated and -triggered host defense against the accidental human pathogen *Legionella pneumophila.*

## Use of the “Accidental” Pathogen *L. pneumophila* as a Model to Understand Effector-Mediated Immune Responses

Several mechanisms of effector-mediated immunity have been uncovered by studying the accidental human pathogen *Legionella pneumophila*. *Legionella* spp. are Gram-negative intracellular bacteria that are ubiquitous in aquatic and soil environments, where they parasitize free-living protozoa (Rowbotham, 1980; Fliermans et al., 1981; Barbaree et al., 1986). Anthropomorphic fresh-water environments such as cooling towers, water fountains and any system that allows for aerosolization of water droplets, have potential to be the source of *Legionella* infection, collectively termed legionellosis (Barbaree et al., 1986). Inhalation or aspiration of *L. pneumophila* can result in an inflammatory pneumonia called Legionnaires’ disease, which is fatal in ~10% of cases (Soda et al., 2017). Legionnaires’ disease primarily affects elderly and immunocompromised individuals and was named for the initial outbreak, which occurred at the 1976 American Legion Convention in Philadelphia (Fraser et al., 1977; McDade et al., 1980). In immunocompetent individuals, *L. pneumophila* can cause a mild self-limiting flu-like illness called Pontiac Fever (Glick et al., 1978). Legionellosis is a consequence of *L. pneumophila* replication within alveolar macrophages (Nash et al., 1984; Friedman et al., 2002); however, the infection is readily cleared by innate immune responses *in vivo*, owing in part to orchestrated production of pro-inflammatory cytokines (Shin, 2012; Liu et al., 2020). The opportunistic colonization of built freshwater environments, rarity of person-to-person transmission and susceptibility to innate immune responses has led to description of *L. pneumophila* as an “accidental pathogen” (Borges et al., 2016; Boamah et al., 2017).

Virulence strategies evolved by *L. pneumophila* to parasitize free-living protozoa have conferred the ability to replicate within mammalian macrophages (Park et al., 2020). Upon phagocytosis, *L. pneumophila* rapidly remodels its vacuole to prevent lysosomal degradation and establish an intracellular replicative niche called the *Legionella* containing vacuole (LCV) (Horwitz, 1983). For biogenesis of LCV and intracellular replication, *L. pneumophila* employs over three hundred individual effector proteins translocated into host cells by a Dot/Icm type IVB secretion system (T4SS) (Berger and Isberg, 1993; Zhu et al., 2011; Ensminger, 2016). *L. pneumophila* encodes the largest arsenal of translocated effector proteins identified to date, due to its broad and diverse tropism for free-living protozoa (Park et al., 2020). Armed with these effectors, *L. pneumophila* proliferates to high numbers within host phagocytes. Effectors are essential for biogenesis of the LCV and intracellular replication through facilitating nutrient acquisition and prevention of lysosomal degradation. However, several *L. pneumophila* effectors that perform these essential functions paradoxically amplify pro-inflammatory immune responses in macrophages. Thus, *L. pneumophila* has become a useful model pathogen to delineate mechanisms of effector-mediated immune detection and clearance. *Legionella* have also served as a valuable model to study molecular basis of inflammasome activation; however, this aspect of *Legionella* biology has been reviewed previously and will not be discussed here (Mascarenhas and Zamboni, 2017).

Below, we discuss mechanisms of effector-mediated immune defense against *L. pneumophila* and the potential for effector-mediated immunity to be harnessed for development of novel therapeutics to combat infectious diseases.

## *L. pneumophila* Effector-Mediated Translation Inhibition Enhances Macrophage Inflammatory Responses

Effector-mediated host protein translation inhibition, a virulence strategy employed by multiple pathogens, enhances inflammatory signaling in *L. pneumophila* infected macrophages (Fontana et al., 2011; Barry et al., 2013). To replicate intracellularly, *L. pneumophila* is reliant on host-derived amino acids (George et al., 1980; Bruckert et al., 2013; Price et al., 2014; Schunder et al., 2014). Since free amino acid levels are tightly regulated in eukaryotic cells, *L. pneumophila* utilizes several effectors to facilitate acquisition of amino acids from host cells. The effectors Lgt1-3, SidI, SidL, LegK4, and RavX collectively inhibit host protein translation [recently reviewed in (Belyi, 2020)]. The mechanisms by which RavX, SidL, and SidI inhibit translation have not been fully elucidated. However, Lgt1-3 glycosylation of the host translation elongation factor eEF1A on a conserved Ser residue inhibits host polypeptide elongation (Belyi et al., 2006; Belyi et al., 2008) and LegK4 impairs polypeptide refolding through phosphorylation of host Hsp90 (Moss et al., 2019). SidI interacts with eEF1A and eEF1Bγ; however, this interaction is not sufficient for translation inhibition (Shen et al., 2009; Joseph et al., 2020). The collective activity of this redundant family of effectors enhances the inflammatory response to *L. pneumophila* (Shin and Roy, 2008; Barry et al., 2013).

**Figure 1:**
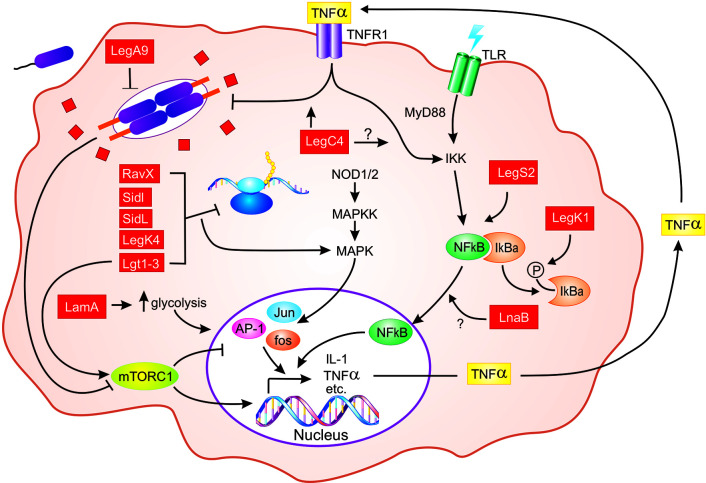
Schematic representation of *L. pneumophila* effector-mediated host defense in macrophages. From the LCV, *L. pneumophila* (purple) translocates hundreds of individual effector proteins (red squares/rectangles) into the host cytosol through the Dot/Icm T4SS (orange). Multiple effectors inhibit host translation elongation (RavX, SidI, SidL, LegK4, and Lgt1-3), which results in activation of MAPK signaling and pro-inflammatory cytokine expression [AP-1 (Jun, Fos)]. The activity of Lgt1-3 also activates the mTORC1 complex, which results in downregulation of pro-inflammatory genes. However, in macrophages, mTOR signaling is attenuated by detection of pathogen-derived molecules. Activation of NF-κB downstream of PRR (TLRs shown) engagement is enhanced by LegS2, LnaB, and LegK1, the latter of which phosphorylates IκBα. *L. pneumophila* replication within macrophages is also impaired by LegA9 and LegC4, the latter of which augments cytokine-mediated restriction. Finally, LamA, a recently characterized *L. pneumophila* effector, degrades cellular glycogen, leading to increased aerobic glycolysis and proinflammatory cytokine production. For clarity, the SidE family of effectors and the role of IL-1 production by infected macrophages are not shown. Question marks indicate unknowns. See text for additional details.

Effector-mediated protein translation inhibition synergizes with PAMP-mediated signaling to enhance inflammation in *L. pneumophila*–infected macrophages. Fontana and colleagues originally discovered that activity of the effectors Lgt1-3, SidI, and SidL induced selective mitogen activated protein kinase (MAPK)-mediated upregulation of interleukin (IL)-1α in *L. pneumophila*–infected macrophages (Fontana et al., 2011; Fontana et al., 2012). MAP kinase signaling cascades culminate in activation of the dimeric activating protein (AP-1) transcription factor—made up of Jun and Fos—which facilitates pro-inflammatory gene expression (Fujioka et al., 2004; Hess et al., 2004; Alonso et al., 2018; Gazon et al., 2018). Interestingly, complementation of a *L. pneumophila* mutant lacking *lgt1-3*, *sidL*, and *sidI* with just *lgt-3* is sufficient to restore MAPK activation during infection (**Figure 1**) (Fontana et al., 2012). Translation inhibition results in selective upregulation of IL-1α, which is critical for host defense against *L. pneumophila* (Barry et al., 2013; Copenhaver et al., 2015; Mascarenhas et al., 2015). The selective upregulation of *Il1a* is a consequence of mRNA superinduction, a phenomenon whereby increased *de novo* transcription of specific genes overcomes bacterial blockade of protein translation and initiates a pro-inflammatory response (Barry et al., 2017). This selective production of IL-1α by infected macrophages results in amplification of pro-inflammatory cytokine production by uninfected translation-competent bystander cells (Copenhaver et al., 2015; Liu et al., 2020) (see below). Translation inhibition also occurs *via* an effector-independent mechanism, which may be a consequence of metabolic reprogramming (Barry et al., 2017; Price et al., 2020) (see below). However, effector-mediated restriction of host protein translation, which liberates amino acids for use by *L. pneumophila* (De Leon et al., 2017), contributes to a highly orchestrated pro-inflammatory response in accidental hosts and is an example of canonical ETI.

## *L. pneumophila* Effector-Mediated Translation Inhibition Impacts Mechanistic Target of Rapamycin (mTOR) Signaling

Modulation of host protein translation also impacts activity of the mechanistic target of rapamycin (mTOR). mTOR is central to many cellular processes and regulates host amino acid metabolism, where availability and dearth of amino acids results in activation or inactivation of mTOR signaling, respectively [**Figure 1**; reviewed in (Condon and Sabatini, 2019)]. Several viral pathogens and protozoan parasites, such as *Leishmania*, have evolved to directly target this pathway and its processes for their own benefit (Buchkovich et al., 2008; Jaramillo et al., 2011; Leroux et al., 2018).

Recent work has revealed the central, albeit complex, role of mTOR in *L. pneumophila* pathogenesis and host defense. Ivanov and Roy initially reported that macrophages detect cytosolic “pathogen signatures,” which results in suppression of mTOR and selective production of pro-inflammatory cytokines and independently of translocated effectors (Ivanov and Roy, 2013). Concomitantly, *L. pneumophila* virulence is attenuated in the lungs of mice with mTOR-deficient macrophages. However, subsequent studies uncovered a role for effectors in mTOR regulation during *L. pneumophila* infection (Abshire et al., 2016; De Leon et al., 2017). The mTORC1 complex (a multiprotein complex containing mTOR) is both suppressed and activated by distinct families of *L. pneumophila* effectors (De Leon et al., 2017). Translation inhibition, through the activity of the Lgt effector family (see above), and consequent increases in free amino acids, activates mTORC1 in macrophages. However, mTORC1 is suppressed through ubiquitination and suppression of Rag GTPases by the SidE effector family (SidE/SdeABC), which also inhibit host protein translation (De Leon et al., 2017). Thus, the SidE family of effectors may prevent mTORC1 sensing amino acids that are liberated downstream of Lgt1-3 activity. In macrophages, mTOR activation by the Lgts is downstream of potent translation inhibition. As discussed above, inhibition of protein translation in macrophages results in selective production of a subset of pro-inflammatory mediators, such as IL-1α. Thus, inhibition of mTOR would contribute to selective production of cytokines that orchestrate a robust inflammatory response in the lung through engagement of bystander cells (see below) (Ivanov and Roy, 2013; Copenhaver et al., 2015; Barry et al., 2017; Liu et al., 2020). Together, these studies collectively emphasize the central and complex role of mTOR in *Legionella* pathogenesis and the inflammatory response elicited in accidental hosts.

## An Effector-Mediated Strategy for Replication in Amoebae Leads to Pro-Inflammatory Macrophage Restriction

*L. pneumophila* is ubiquitous in freshwater environments where it parasitizes and replicates within unicellular eukaryotes, including amoebae (Molmeret et al., 2005; Albert-Weissenberger et al., 2006). When environmental conditions are not optimal for growth and survival, amoeboid trophozoites undergo encystation, a condition where the amoeba turns into a metabolically inactive cyst containing cellulose rich cell wall that is resistant to hostile environmental conditions (Moon and Kong, 2012; Aqeel et al., 2013). Although *L. pneumophila* survives in amoebal cysts, encystation is restrictive to intracellular replication (Kilvington and Price, 1990; Bouyer et al., 2007). Prior to encystation, amoebae accumulate glycogen, which is used for biogenesis of the characteristic cellulose-rich cell wall (Weisman et al., 1970; Fouque et al., 2012; Moon and Kong, 2012; Schaap and Schilde, 2018).

To maintain amoebae as replication-permissive trophozoites, *L. pneumophila* utilizes the effector LamA, an amylase that catalyzes glycogenolysis to limit glycogen accumulation in infection amoebae (Price et al., 2020). LamA alone is not required for *L. pneumophila* replication in the natural hose *Acanthamoeba polyphaga*, likely due to functional redundancy with other effectors (Ghosh and O’Connor, 2017; Park et al., 2020). However, other natural host amoebae were not examined in this study. Thus, it is tempting to speculate that LamA activity is individually important in other species, such as *A. castellanii*, in which encystation is highly restrictive to *L. pneumophila* (Weisman et al., 1970; Bouyer et al., 2007).

LamA-mediated metabolic reprogramming is deleterious to *L. pneumophila* in accidental hosts (Price et al., 2020). Excess cellular glucose results in increased aerobic glycolysis in both *A. polyphaga* and human monocyte-derived macrophages (hMDM) (Price et al., 2020). Aerobic glycolysis in macrophages promotes their activation, M1 polarization and secretion of pro-inflammatory cytokines (Langston et al., 2017). Thus, LamA activity enhances secretion of pro-inflammatory cytokines from hMDMs during infection [**Figure 1** (Price et al., 2020)] and impairs *L. pneumophila* in hMDM through IFN-γ–mediated indolamine-2,3-dioxygenase (IDO) activity (Murray et al., 1989; Price et al., 2020). This result is intriguing since translation inhibition during *L. pneumophila* infection limits production of most cytokines (see above). The authors propose that the amount of IFN-γ produced is sufficient for IDO activation; however, it would be interesting to determine if IFN-γ activation is indeed required for LamA-mediated restriction. In the mouse lung, IL-1α production is severely decreased following infection with a *lamA* mutant compared to wild-type bacteria. However, the *lamA* mutant strain presumably still translocates effector translation inhibitors (see above). Thus, macrophage metabolic reprogramming may contribute to effector-independent translation inhibition and concomitant inflammation (Barry et al., 2017). Moreover, LamA-mediated macrophage activation is a direct consequence of its enzymatic activity, distinguishing this response from canonical ETI.

## Effector-Mediated Augmentation of Cytokine-Mediated Restriction of *L. pneumophila*

Pro-inflammatory cytokines activate resting macrophages and are critical for restriction of *L. pneumophila* in mammalian hosts (Archer and Roy, 2006). The effector LegC4 was initially identified in a high-throughput forward genetic screen for individual effectors that impact *L. pneumophila* virulence in amoeba and mammalian infection models (Shames et al., 2017; Rolando and Buchrieser, 2018). This screen identified LegC4 as conferring fitness disadvantage on *L. pneumophila* relative to the isogenic parental strain in a mouse model of Legionnaires’ disease but not BMMs *ex vivo* (Shames et al., 2017; Ngwaga et al., 2019). Interestingly, LegC4 is individually important for *L. pneumophila* replication in the natural host, *A. castellanii* (Shames et al., 2017). Further investigation revealed that LegC4 is deleterious to *L. pneumophila* specifically within cytokine-activated macrophages (Ngwaga et al., 2019) (**Figure 1**). In cultured mouse BMMs, LegC4-mediated restriction is contingent on autocrine and paracrine TNF receptor 1(TNFR1)-mediated signaling. However, loss of TNFR1 is insufficient to rescue LegC4-mediated replication defects in the mouse lung, likely due to LegC4-mediated exacerbation of IFN-γ-mediated restriction (Ngwaga et al., 2019). LegC4 additionally enhances secretion of several pro-inflammatory cytokines, including IL-12, IL-6, and TNF-α, from *L. pneumophila*–infected BMMs despite global translation inhibition (Shames et al., 2017; Ngwaga et al., 2019). Whether LegC4-mediated increases in cytokine production from *L. pneumophila*–infected macrophages is due to enhanced transcription or translation is unknown. Revealing the influence of LegC4 on production of IL-1α, TNF-α, and IFN-γ in the lung will provide a foundation for understanding the mechanism of LegC4-mediated restriction.

Interestingly, LegC4 is also augments cytokine-mediated restriction of *L. longbeachae*, and the second leading cause of Legionnaires’ disease globally (Gobin et al., 2009). *L. longbeachae* is lethal to mice and is reliant on a Dot/Icm T4SS for intracellular replication; however, *L. longbeachae* does not encode a homolog of *legC4* (Cazalet et al., 2010; Wood et al., 2015; Massis et al., 2016). LegC4 is sufficient to attenuate *L. longbeachae* replication within BMMs activated with either TNF-α or IFN-γ (Ngwaga et al., 2019), demonstrating that LegC4-mediated restriction is not specific to *L. pneumophila*. LegC4—like LamA—enhances *L. pneumophila* virulence in a natural host, but its activity in macrophages is deleterious. The mechanism by which LegC4 impacts *L. pneumophila* fitness in natural and accidental hosts, respectively, and its potential to enhance cytokine-mediated restriction of other intracellular pathogens are currently under investigation in our lab.

## Effector-Mediated Activation of Inflammatory Gene Expression and Autophagy

Many bacterial pathogens actively attenuate inflammatory signaling by restricting activation of the NF-κB transcription factor (Brodsky and Medzhitov, 2009). However, NF-κB is activated in mammalian cells infected with *L. pneumophila*. Within *L. pneumophila*–infected cells, NF-κB activation occurs in two waves; effector-independent TLR-dependent activation when bacteria first make contact with host cells and effector-mediated activation after several hours of infection (Losick and Isberg, 2006; Asrat et al., 2014). Several *L. pneumophila* effectors contribute to NF-κB activation in mammalian cells (**Figure 1**). LegK1 is a eukaryotic-like serine/threonine kinase that phosphorylates the inhibitor of κBα (IκBα), which results in nuclear localization of NF-κB and consequent upregulation of pro-inflammatory and pro-survival gene (Ge et al., 2009; Rahman and McFadden, 2011). However, LegK1-mediated NF-κB activation occurs only upon ectopic expression of *legK1* in epithelial cells (Ge et al., 2009). LnaB enhances NF-κB-mediated gene expression by an unknown mechanism following ectopic expression and during *L. pneumophila* infection of epithelial cells (Losick et al., 2010). LegK1 does not contribute to NF-κB activity in *L. pneumophila*–infected epithelial cells (Losick et al., 2010), which raises the possibility that this phenotype is a consequence of dose-dependent effect, or mislocalization due to ectopic expression. Neither *lnaB* nor *legK1* are required for *L. pneumophila* replication in mouse macrophages individually or in combination (Losick et al., 2010). Since amoebae lack NF-κB signaling components, direct effector-mediated activation of this pathway is perplexing. It is possible that IκBα phosphorylation by LegK1 is due promiscuous enzymatic activity and/or presence highly conserved target motifs. Identification of LegK1 substrates in amoebae would shed light on this possibility. Effector-mediated NF-κB activation enhances *L. pneumophila* survival in macrophages through prevention of premature apoptosis but also results in expression of pro-inflammatory cytokines, including IL-1α (Losick and Isberg, 2006). NF-κB plays a multifaceted role in *L. pneumophila* infection of accidental hosts, but the evolutionary basis for its activation has yet to be elucidated.

Autophagy is central to cell-autonomous restriction of intracellular bacterial pathogens in phagotrophs. *L. pneumophila* has evolved several effectors capable of regulating host autophagy and two, LegS2 and LegA9, are deleterious to *L. pneumophila* in accidental hosts (Khweek et al., 2016; Rolando et al., 2016; Sherwood and Roy, 2016). LegS2 is a mitochondria-targeted sphingosine-1-phosphate lyase that restricts *L. pneumophila* replication in macrophages, suppresses autophagy, and enhances NF-κB activation (Degtyar et al., 2009; Khweek et al., 2016; Rolando et al., 2016). Suppression of starvation-induced autophagy by LegS2 is facilitated by modulation of host sphingosine metabolism (Rolando et al., 2016), but whether amplification of NF-κB is linked to LegS2-mediated sphingosine metabolism and suppression of autophagy is unknown. LegA9 enhances *L. pneumophila* macrophage clearance by upregulating autophagy in BMMs (Khweek et al., 2013); however, further investigation is required to define the mechanism by which LegA9 augments *L. pneumophila* macrophage clearance.

## Consequences of Effector-Mediated Immunity in a Mouse Model of Legionnaires’ Disease

*L. pneumophila* replicates robustly in macrophages derived from permissive mice but is efficiently cleared from the lung just days after infection. Restriction of *L. pneumophila* in the mouse lung is due to a rapid and robust pro-inflammatory response orchestrated through engagement of multiple cell types (Blanchard et al., 1987; Blanchard et al., 1989; Brieland et al., 1998; Archer and Roy, 2006). *L. pneumophila*–infected alveolar macrophages are poor producers of TNF-α, IL-6, and IL-12 *in vivo* due to effector-mediated translation inhibition (Copenhaver et al., 2014; Copenhaver et al., 2015). However, selective upregulation of IL-1α by infected translation-impaired cells ultimately results in pro-inflammatory cytokine production by uninfected bystander cells, namely Ly6C^hi^ monocytes and neutrophils (Copenhaver et al., 2015; Barry et al., 2017; Casson et al., 2017). A central role for IL-1α in immune defense against *L. pneumophila* has been well established and is contingent on MyD88-mediated signaling (Barry et al., 2013; Asrat et al., 2014; Copenhaver et al., 2015; Mascarenhas et al., 2015). However, the mechanism by which IL-1α facilitates bacterial clearance *in vivo* was only recently uncovered. IL-1α produced by infected alveolar macrophages engages IL-1R on alveolar epithelial cells, which in turn secrete granulocyte colony stimulating factor (GM-CSF) (Liu et al., 2020). Consequent GM-CSF signaling in inflammatory monocytes upregulates aerobic glycolysis leading to pro-inflammatory cytokine production (Liu et al., 2020). This work exemplifies how effector-driven virulence mechanisms, such as translation inhibition, trigger a highly orchestrated inflammatory response to *L. pneumophila* in the lung.

## Harnessing Effector-Mediated Immunity to Combat Bacterial Infection

Antimicrobial resistance comprises a major global public health challenge. Thus, to control the emergence and spread of antimicrobial-resistant pathogens, innovative therapeutic strategies are desperately needed. Anti-virulence therapy a promising alternative approach to combat resistant pathogens *via* targeting virulence pathways of the pathogen (Rasko and Sperandio, 2010; Martínez et al., 2019). As pathogen-centric therapeutics are susceptible to evolution of resistance, host-centric therapeutics are an attractive alternative to control bacterial infection. Information gleaned from effector-mediated immune response and the effectors themselves have potential to combat infection by regulated amplification of host immunity.

Bacterial products, including effectors, modulate host immunity. Bacterial PAMPs are promising innate immunologicals, but whether immune-activating effectors can be harnessed directly or indirectly to combat infectious diseases has not been investigated. CpG oligodeoxynucleotides (ODN), a TLR9 agonist, have been used as vaccine adjuvants and can amplify immune responses to multiple bacterial, parasitic and viral pathogens, including *Leishmania major*, *Mycobacterium tuberculosis*, *Francisella tularensis* and, more recently, SARS-CoV-2, the etiological agent of COVID-19 (Zimmermann et al., 1998; Elkins et al., 1999; Juffermans et al., 2002; Scheiermann and Klinman, 2014; Oberemok et al., 2020). Moreover, multiple effectors from well-adapted human pathogens that dampen host immunity have attracted attention as potential drug candidates for the treatment of inflammatory diseases (Rüter and Hardwidge, 2014).

Use of effectors that amplify immunity directly into host cells as possible host-specific therapeutics has not been evaluated. Effector-mediated subversion of host homeostasis triggers ETI but perturbation of host cellular processes poses a major challenge. Global inhibition of translation as a means to enhance anti-microbial immunity would be impractical. However, based on insight from the response to ETI, treatment with IL-1α could initiate an early and robust immune response in the lung against diverse pathogens (see above). The effector LegC4 is an intriguing candidate for host-specific therapeutics based on its ability to amplify cytokine-mediated pathogen restriction. However, further investigation is required to determine the mechanisms of LegC4 function within immune cells and if immune-activating effectors can be harnessed as host-specific therapeutics.

To exert their functions, effectors require access to the cytosol of host cells. Current use of effectors as therapeutics is accomplished by autonomous translocation into host cells *via* fusion to cell-penetrating peptides (Rüter and Schmidt, 2017). However, the N-terminal domain of anthrax lethal factor (LFn), when co-delivered with the anthrax protective antigen, facilitates translocation of cargo protein directly into mammalian cells (Rabideau and Pentelute, 2016). LFn fusion has been successfully utilized to deliver bacterial proteins into host cells and mice (Kofoed and Vance, 2011; Shi et al., 2015). In addition, nanoparticles can be used for specific delivery of nucleic acids for orthologous expression of effectors within target cells or direct delivery of protein cargo (Avila et al., 2016; Rincon-Restrepo et al., 2017).

Thus, potential exists for bacterial effectors to function as therapeutic agents. Effector based therapeutics offer several advantages over conventional biologics such as autonomous translocation, enhanced specificity, efficacy at low concentrations, targeted and topical applications, comparatively fewer side effects, cost-effective and stability at variable pH and temperatures. Moreover, further understanding of novel effectors, mechanisms of effector-mediated immunity, and the development of selective delivery mechanisms offers potential for improved combinatorial therapeutics in the future.

## Conclusions

In addition to sensing pathogens through PRRs, the mammalian immune system has developed additional mechanisms to detect the activity of virulence factors secreted by pathogens. Effector-mediated immunity facilitates detection and/or enhanced clearance of pathogens. This additional mechanism to detect pathogens is important because pathogenic microorganisms have evolved ways to avoid, modulate and hide from the first line of immune defense offered by triggering of PRRs. As an accidental human pathogen, *L. pneumophila* continues to serve as a useful model used to study innate immune mechanisms without the complications of evasion strategies used by mammalian-adapted pathogens. *L. pneumophila* has provided valuable insight into mechanisms of innate immune defense against intracellular bacterial pathogens, including how effector-mediated virulence strategies trigger inflammation. Further investigation of *L. pneumophila* effector function will undoubtedly reveal yet additional mechanisms by which cells of the innate immune system restrict intracellular pathogens. Exploiting effector-mediated immunity to elicit pathogen-centric immunotherapeutics may provide additional treatment or prevention strategies against antimicrobial resistant pathogens.

## Author Contributions

SS conceived the idea for the manuscript. TN, DC, and SS wrote the paper. All authors contributed to the article and approved the submitted version.

## Funding

The Shames Lab is supported by an NIH NIGMS COBRE Research Project Grant (P20GM130448; SS), an NIH Kansas-INBRE Postdoctoral Fellowship (P20GM103418; DC), U.S. Department of Agriculture Research Service project (#3020-43440-001-00D; SS) and institutional start-up funds from Kansas State University (SS).

## Conflict of Interest

The authors declare that the research was conducted in the absence of any commercial or financial relationships that could be construed as a potential conflict of interest.
